# Enhancing multimodal survival prediction: tri-modal learning with clinical knowledge integration via state space models

**DOI:** 10.3389/fonc.2026.1825286

**Published:** 2026-08-04

**Authors:** Yijiang Ding, Yuanwei Jing, Wanhan Zhang

**Affiliations:** 1 Faculty of Engineering, University of Malaya, Kuala Lumpur, Malaysia; 2 School of Electronics and Information, Xi’an Polytechnic University, Xi’an, China

**Keywords:** cancer survival analysis, computational pathology, Mamba, multimodal learning, multiple instance learning

## Abstract

Accurate survival prediction is crucial for precision oncology, yet it faces challenges due to the neglect of clinical priors and high computational complexity. We propose TriBind-Mamba, a tri-modal framework integrating Clinical Knowledge Prompting (CKP) and selective State Space Models (SSMs). By transforming structured clinical records into semantic narratives using Large Language Models (LLMs), our model provides high-level context for morphological and molecular features. TriBind-Mamba efficiently processes gigapixel whole slide images and transcriptomic profiles with linear complexity, achieving state-ofthe-art performance (Overall C-index of 0.664) across five TCGA cohorts while significantly reducing computational overhead. Interpretability is enhanced by integrating human-readable clinical knowledge prompts, biologically meaningful pathway-level transcriptomic tokens, and WSI attention heatmaps that project model-derived importance scores back onto histopathological regions. These analyses suggest that TriBind-Mamba focuses on prognostically relevant malignant areas, providing a more transparent basis for multimodal survival prediction.

## 1 Introduction

Survival analysis, also known as time-to-event analysis, focuses on the probabilistic assessment of experiencing a specified event, such as mortality or recurrence, within a clinical setting before a given time under both uncensored and right-censored data (1–3). In the realm of cancer management, survival prediction serves as a cornerstone of precision oncology, enabling the quantification of prognostic risks and assisting clinicians in formulating more targeted diagnostic decisions, patient stratification, and treatment planning (4–6).

With the rapid advancement of digital pathology and high-throughput sequencing, survival prediction has evolved from traditional qualitative clinical assessments to data-driven quantitative evaluations. Early deep learning research predominantly focused on unimodal approaches, leveraging either histological whole slide images (WSIs) (7–9) or transcriptomic data (10, 11). Image-based unimodal methods typically employ Multiple Instance Learning (MIL) frameworks to process gigapixel WSIs, capturing morphological heterogeneity within the tumor microenvironment for risk stratification. However, relying solely on morphological features often fails to reveal the underlying deep biological regulatory mechanisms. Conversely, genomics-based unimodal methods provide a global landscape of cellular states but frequently neglect the spatial context of tissue structure. Moreover, the high-dimensional and sparse nature of genomic features often leads to model overfitting.

To overcome these unimodal limitations, numerous multi-modal survival prediction frameworks have emerged, aiming to enhance predictive accuracy by fusing histological morphological features with genomic profiles (12–15). Early efforts in multi-modal integration focused on early fusion methods to combine disparate modal features (16). However, these methods often failed to capture fine-grained interactions between modalities. Consequently, more sophisticated architectures, such as MCAT (14) and SurvPath (16), have been proposed to model dense interactions between histology patches and biological pathways via attention mechanisms. Despite these advancements, current multi-modal approaches still face three critical problems: 1) Neglect of clinical priors. Existing frameworks often overlook the intrinsic value of structured clinical records (e.g., age, tumor stage, and grade) as high-level prior knowledge, failing to fully exploit their semantic associations with biological phenotypes. 2) High computational cost. Traditional Transformer-based architectures suffer from quadratic computational complexity relative to sequence length, imposing significant overhead when processing ultra-long pathological sequences and extensive genomic markers (17). 3) Inadequate feature differentiation. Maintaining modality-specific differences while exploring inter-modal similarities remains a fundamental challenge. Existing models frequently prioritize modality-invariant features (e.g., shared or synergistic representations) while neglecting the importance of modality-specific nuances. This often results in a “modality collapse” where the model over-relies on a single dominant modality, undermining overall robustness.

To address these problems, we propose a novel tri-modal binding learning framework integrated with clinical knowledge prompting, named TriBind-Mamba. Specifically, To leverage high-level clinical priors, we introduce Clinical Knowledge Prompting (CKP) as a third modality. By utilizing Large Language Models (LLMs), structured clinical records are transformed into semantically rich text descriptions, bridging the gap between clinical priors and biological phenotypes in a shared semantic space. We design a Binding Learning mechanism to facilitate cross-modal interactions, comprising bimodal and trimodal binding strategies. To efficiently process these bound features, we introduce the Cross Modal Binding Mamba and Trimodal Binding Learning Mamba modules. By replacing the traditional multi-head attention architecture with a selective state space model (SSM) (18) possessing linear complexity, our framework achieves multi-grained deep interactions across tri-modal sequences while significantly reducing computational overhead. To effectively balance modality-specific and modality-invariant representations, we implement a joint optimization strategy consisting of task loss, similarity loss, and dissimilarity loss. This strategy ensures robust alignment and discrimination of modal features, preventing redundant information from dominating the prediction. Extensive evaluations on five public TCGA datasets demonstrate that our proposed framework significantly enhances survival prediction accuracy while substantially reducing computational complexity, proving its immense potential for clinical decision support.

The contributions of this paper are summarized as follows:

- We introduce Clinical Knowledge Prompting as a novel modality, utilizing LLMs to translate structured clinical data into a semantic space for enhanced prognostic modeling.
- We propose a novel tri-modal binding learning framework, which integrates Mamba-based linear-time sequence modeling into a multi-modal binding framework for efficient tri-modal interaction.
- We design a robust joint loss function to explicitly distinguish between modality-invariant and modality-specific features, ensuring balanced and stable multi-modal fusion.
- Our method achieves state-of-the-art performance across multiple cancer types with a lower computational footprint compared to existing Transformerbased models.

## 2 Related works

### 2.1 State space models

State Space Models (SSMs) have recently emerged as a potent alternative to Transformers for sequence modeling, particularly when dealing with long-range dependencies. Built upon the foundation of continuous-time systems, modern SSMs like the Structured State Space Sequence model (S4) (19) utilize a principled discretization approach to achieve both parallelizable training and efficient inference. By employing a structured representation of the state transition matrix, SSMs bypass the quadratic complexity inherent in the self-attention mechanism of Transformers, offering *O*(*L*log*L*) or *O*(*L*) computational complexity relative to sequence length *L*.

The evolution from S4 to the Mamba architecture (18) introduced a selective mechanism (S6) that allows the model to compress information into a hidden state adaptively based on the input content. This “selective” property enables Mamba to capture context-dependent features similar to Transformers while maintaining the linear scaling required for high-resolution medical data. In the medical domain, Mamba has shown remarkable potential in tasks such as image classification (20), image segmentation (21), and long-sequence pathological analysis (17). In this work, we extend the application of Mamba to tri-modal survival prediction, leveraging its efficiency to process ultra-long histology sequences and complex pathway profiles simultaneously.

### 2.2 Prompt learning

Prompt learning was initially popularized in natural language processing (NLP) and aims to leverage the vast knowledge embedded in Large Language Models (LLMs) by reformulating downstream tasks into a format based on prompts (22, 23). The core paradigm involves using prompts in the form of either hard discrete text or soft learnable embeddings to steer the pre trained model representations toward specific objectives without the need for extensive fine tuning.

In the context of multimodal learning, strategies based on prompts have been successfully utilized to bridge the gap between heterogeneous data sources such as text and images in CLIP (24). Recent advances in clinical research have explored the use of LLMs to transform structured medical records into semantically rich narratives which serve as knowledge prompts to provide high level context for lower level biological data (25, 26). Our TriBind-Mamba framework builds on this concept by introducing Clinical Knowledge Prompting (CKP) which utilizes narratives synthesized by LLMs as a third modality to provide prognostic anchors for survival prediction.

### 2.3 Multimodal survival analysis

Multimodal survival analysis integrates disparate data sources such as Whole Slide Images (WSIs) genomics and clinical records to provide a holistic assessment of patient risk (12–14). Traditional deep learning models for survival prediction often treat these modalities independently or use late fusion strategies that fail to capture cross modal synergies (27). To address this attention based bimodal frameworks like MCAT (14) and SurvPath (16) were developed to model dense interactions between histology patches and transcriptomic biological pathways.

Despite their performance gains these models face significant challenges. Transformer based architectures like SurvPath exhibit quadratic complexity making them computationally prohibitive for ultra long patch sequences (28). Furthermore existing frameworks often neglect the intrinsic prognostic value of structured clinical priors which are frequently used as secondary filters rather than integrated features. Most importantly the problem of modality collapse in which the model relies excessively on a single dominant modality remains pervasive. TriBind-Mamba addresses these gaps by utilizing Mamba for linear time sequence modeling and integrating clinical knowledge as a primary modality via prompting as well as implementing a binding learning strategy with joint optimization to ensure balanced feature differentiation.

## 3 Methods

### 3.1 State space models

The State Space Model (SSM) architecture serves as the foundation for modeling long-range dependencies in high-dimensional medical sequences. Fundamentally, an SSM maps a one-dimensional input stimulus x(t)∈ℝ to an output response y(t)∈ℝ through an intermediate latent state $h\left(t\right)\in {\mathbb{R}}^{N}$. This continuous-time evolution is parameterized by the quadruple (Δ, *A*, *B*, *C*) and defined by the following linear ordinary differential equations:

(1) $$h^{\prime }\left(t\right)=Ah\left(t\right)+Bx\left(t\right),\;y\left(t\right)=Ch\left(t\right)$$

where $A\in {\mathbb{R}}^{N\times N}$ represents the state transition matrix, and $B\in {\mathbb{R}}^{N\times 1},C\in {\mathbb{R}}^{1\times N}$ represent the projection parameters. To implement the system in digital frameworks, the continuous parameters are transformed into discrete counterparts $\left(\bar{A},\bar{B}\right)$ via a timescale parameter Δ. Utilizing the zero-order hold (ZOH) discretization method, the conversion is defined as:

(2) $$\bar{A}=\exp(\text{Δ}A),\;\bar{B}=\left(\text{Δ}A{)}^{-1}\left(\exp(\text{Δ}A\right)-I\right)\cdot \text{Δ}B$$

Post-discretization, the system facilitates efficient auto-regressive inference through a recurrent formulation:

(3) $$h_{t}=\bar{A}h_{t-1}+\bar{B}x_{t},\;y_{t}=Ch_{t}$$

For large-scale training on high-dimensional datasets, the model can be reformulated as a global convolution. Given an input sequence *x* of length *M*, the output is derived using a structured convolutional kernel $\bar{K}\in {\mathbb{R}}^{M}$:

(4) $$\bar{K}=\left(C\bar{B},C\bar{AB},\ldots ,C{\bar{A}}^{M-1}\bar{B}\right),\;y=x*\bar{K}$$

The Mamba architecture improves upon the S4 foundation by introducing a data-dependent selection mechanism (S6). Unlike static SSMs where parameters remain constant across the sequence, Mamba enables *B*, *C*, and Δ to be functions of the input *x_t_*. This selective mechanism allows the model to adaptively propagate or forget information based on the specific sequence content, effectively emulating the selective attention of Transformers while retaining the linear computational complexity required for processing gigapixel whole slide images and extensive transcriptomic profiles.

### 3.2 Framework overview

**Figure 1:**
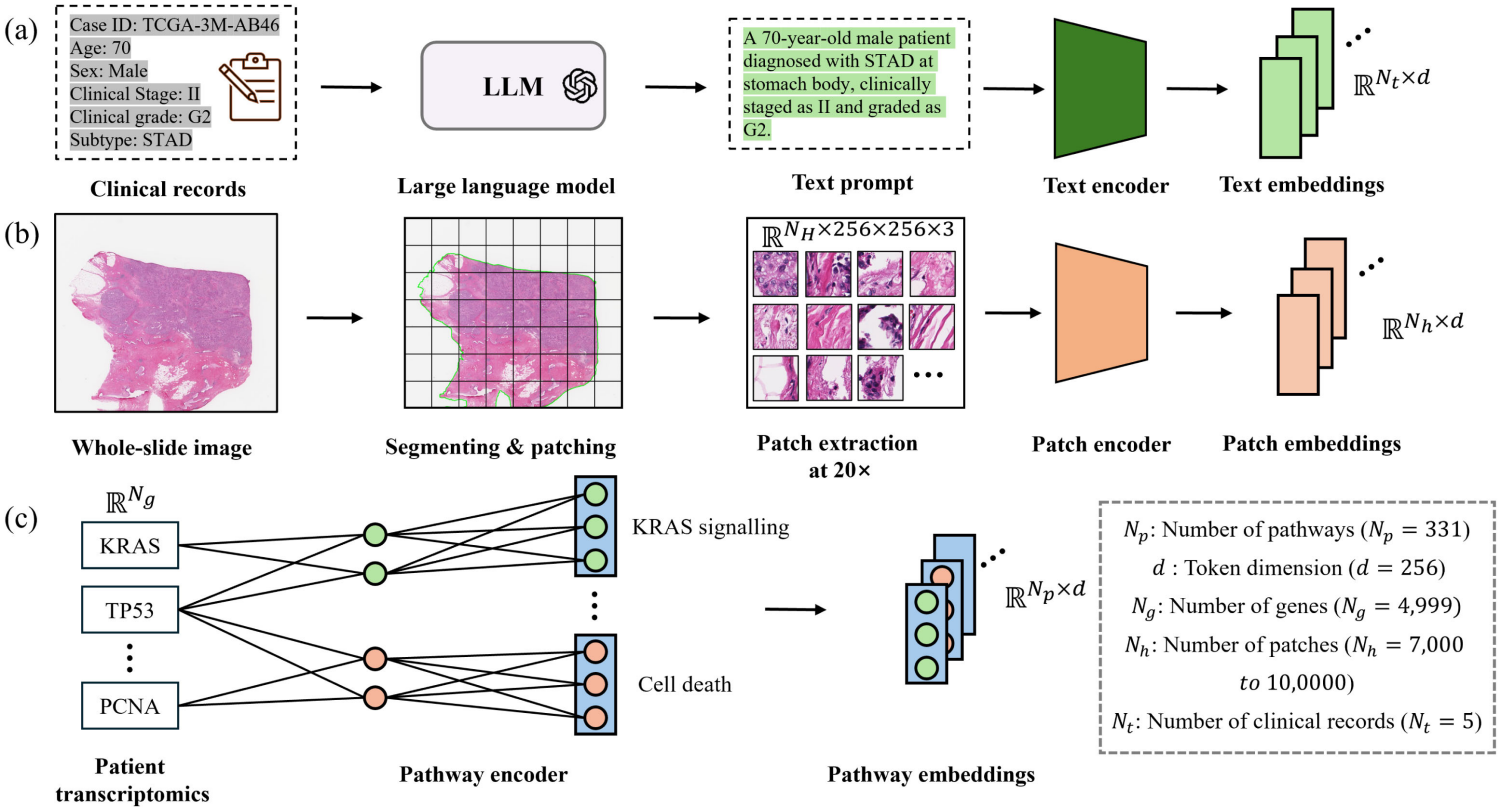
The proposed data construction and feature extraction pipeline for TriBind-Mamba. **(a)** Clinical records are expanded into semantic narratives via LLM and encoded into text prompts. **(b)** Histology WSIs are segmented and transformed into patch tokens using a pathology foundation model. **(c)** Transcriptomic profiles are mapped into biologically interpretable pathway tokens through a sparse MLP (S-MLP) constrained by gene-to-pathway connectivity.

**Figure 2:**
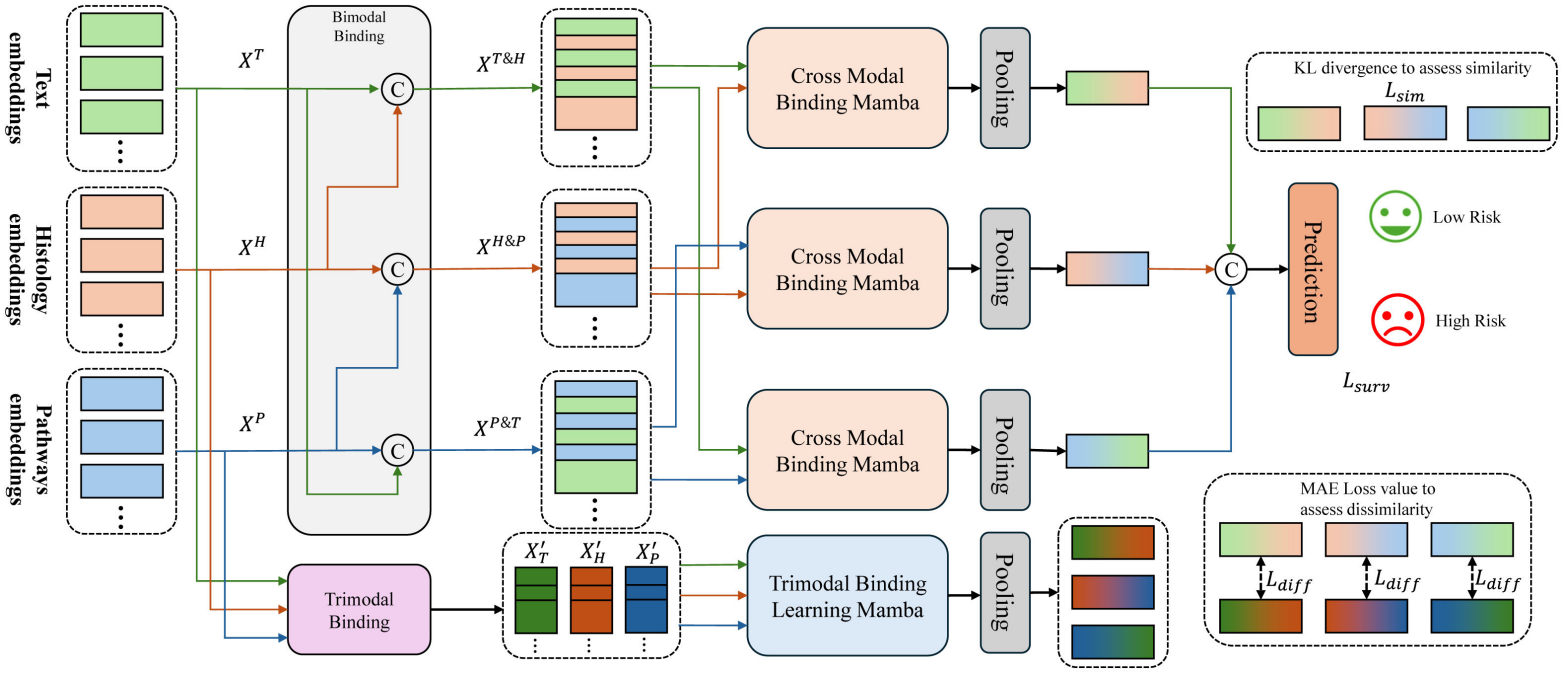
Overview of the TriBind-Mamba architecture. After the multimodal features are integrated through Binding Learning and Mamba encoding, they are used for the survival analysis task.

As illustrated in **Figure 1**, the proposed TriBind-Mamba framework initiates with a multi-scale data construction and feature extraction phase, during which clinical records, whole slide images (WSIs), and transcriptomic data are transformed into three independent embedding spaces. Subsequently, as depicted in **Figure 2**, these latent representations are processed through a hierarchical two-stage binding learning architecture. In the first stage, the model features are explicitly decoupled into two categories: modality-invariant and modality-specific representations. Specifically, modality-specific features encapsulate the inherent characteristics unique to each individual data source, while modality-invariant features represent the shared semantic information across all modalities. In the second stage, we utilize an optimized cross-modal Mamba architecture to further encode the resulting features. This architecture is specifically designed to capture multi-grained interaction representations, effectively bridging fine-grained local features with coarse-grained global contexts across the interacting modalities. To ensure robust and stable representation learning, the entire framework is jointly optimized through a composite loss function. This objective function includes a task-specific survival prediction loss, a similarity loss to align modality-invariant features, and a dissimilarity loss to enforce the distinctiveness of modality-specific features.

### 3.3 Data construction and feature extraction

#### 3.3.1 Clinical knowledge prompting

Clinical variables are routinely used in oncology for patient stratification and prognostic assessment. To incorporate such clinical priors into multimodal survival prediction, we introduce Clinical Knowledge Prompting (CKP), which converts structured clinical records into standardized patient-level clinical narratives. The input variables include demographic information and routinely available diagnostic indicators, such as age, sex, pathological stage, T/N/M category, and histological grade. Survival-related variables, including survival time, censoring status, disease-specific survival labels, recurrence information, and model-derived risk groups, are strictly excluded from the prompt input to avoid potential label leakage.

In the default setting, GPT-4 (29) is used to generate clinical narratives from structured records because of its strong instruction-following ability and robustness in handling heterogeneous clinical fields. However, TriBind-Mamba is not dependent on GPT-4 specifically. The purpose of the language model in CKP is to normalize tabular clinical information into a consistent semantic text format that can be encoded and aligned with histological and transcriptomic representations. Therefore, the CKP module can also be implemented using deterministic rule-based templates or open-source LLMs, as further evaluated in Section 4.4.4. To ensure reproducibility, a fixed prompt template is used for all patients and all cohorts. For missing clinical variables, the corresponding field is retained and explicitly marked as “unavailable,” rather than being removed or imputed. This design preserves a consistent narrative structure across patients and prevents the language model from inferring unobserved clinical information. All clinical narratives are generated before model training and remain fixed during cross-validation. The complete prompt template, input variables, and generation settings are provided in **Appendix 1**, and representative generated examples and the rule-based baseline are provided in **Appendix 2**.

To further minimize leakage risk, the prompt explicitly instructs the language model not to infer prognosis, survival outcome, recurrence, mortality, treatment response, or risk group. We also inspected generated narratives to confirm that they did not contain explicit survival labels, follow-up outcomes, or model-derived risk information. Because all narratives were generated before cross-validation and kept fixed across folds, no fold-specific or label-derived information was introduced during narrative generation.

The generated clinical narrative is encoded using the frozen text encoder of the CONCH foundation model (30). The resulting text representation is projected into the unified hidden dimension, yielding the patient-level clinical representation $X^{T}\in {\mathbb{R}}^{N_{T}\times d}$, where *N_T_* denotes the number of clinical text tokens. This representation is used as the clinical modality and integrated with histology patch tokens and pathway-level transcriptomic tokens in the subsequent binding learning module.

#### 3.3.2 Histology whole slide image representation

For each input Whole Slide Image (WSI), we aim to derive discriminative patch-level representations while preserving the spatial hierarchy of the tumor microenvironment. The process initiates with a tissue-segmentation algorithm to isolate relevant biological regions, ensuring that non-informative background areas are excluded.

The identified tissue regions are decomposed into a set of *N_H_* non-overlapping patches at 20× magnification, denoted as $H^{H}=\left\{h_{1},h_{2},\ldots ,h_{N_{H}}\right\}$. Given the extreme resolution of gigapixel WSIs, patch-level embeddings are pre-extractedto optimize computational efficiency during model training. We utilize the pre-trained image encoder of the CONCH foundation model (30), *f*(·), to map each raw patch into a compact feature vector: *x_i_* = *f*(*h_i_*). These embeddings represent highly compressed morphological features, which are subsequently projected through a learnable linear layer to yield histology patch tokens $X^{H}\in {\mathbb{R}}^{N_{H}\times d}$.

#### 3.3.3 Pathway-aware transcriptomics representation

To capture the intricate hierarchy of cellular biological processes, we utilize biological pathways as fundamental reasoning units. Given transcriptomics measurements $g\in {\mathbb{R}}^{N_{G}}$, we construct pathway-level tokens $X^{P}\in {\mathbb{R}}^{N_{P\times d}}$ by organizing genomic data into a multi-layered structure across gene-, function-, and process-levels.

Specifically, we employ a *sparse* multi-layer perceptron (S-MLP) (31, 32) to map genes to their respective functional genomes, categorized by biological process. The pathway-specific weights *ϕ_i_* generate individual tokens: $x_{i}^{P}=\phi_{i}\left(g_{P_{i}}\right)$, where $g_{P_{i}}$ represents the subset of genes within pathway *P_i_*. The network sparsity is strictly governed by curated gene-to-pathway connectivity to identify specific functionalities of gene sets. The concatenated output is reshaped into $X^{P}\in {\mathbb{R}}^{N_{P\times d}}$, providing a biologically interpretable and learnable representation for downstream multimodal fusion.

### 3.4 Binding learning and binding Mamba module

#### 3.4.1 Binding learning

Given the extracted features $X^{T}\in {\mathbb{R}}^{N_{T}\times d}$, $X^{H}\in {\mathbb{R}}^{N_{H}\times d}$, and $X^{P}\in {\mathbb{R}}^{N_{P}\times d}$, we propose specialized binding strategies, namely Bimodal Binding and Trimodal Binding, to fully leverage the potential for inter-modal interaction within the extracted feature embeddings.

Bimodal Binding: Bimodal Binding combines non-aligned modalities that share modality-invariant features and feeds them into a cross-modal binding Mamba for cross-modal learning. A detailed description of the Mamba structure will be provided in the subsequent section. Here, we use concatenation operations for bimodal binding learning, $X^{T\&H}=\text{CAT}(X^{T},X^{H})$, $X^{H\&P}=\text{CAT}(X^{H},X^{P})$, $X^{P\&T}=\text{CAT}(X^{P},X^{T})$.

**Figure 3:**
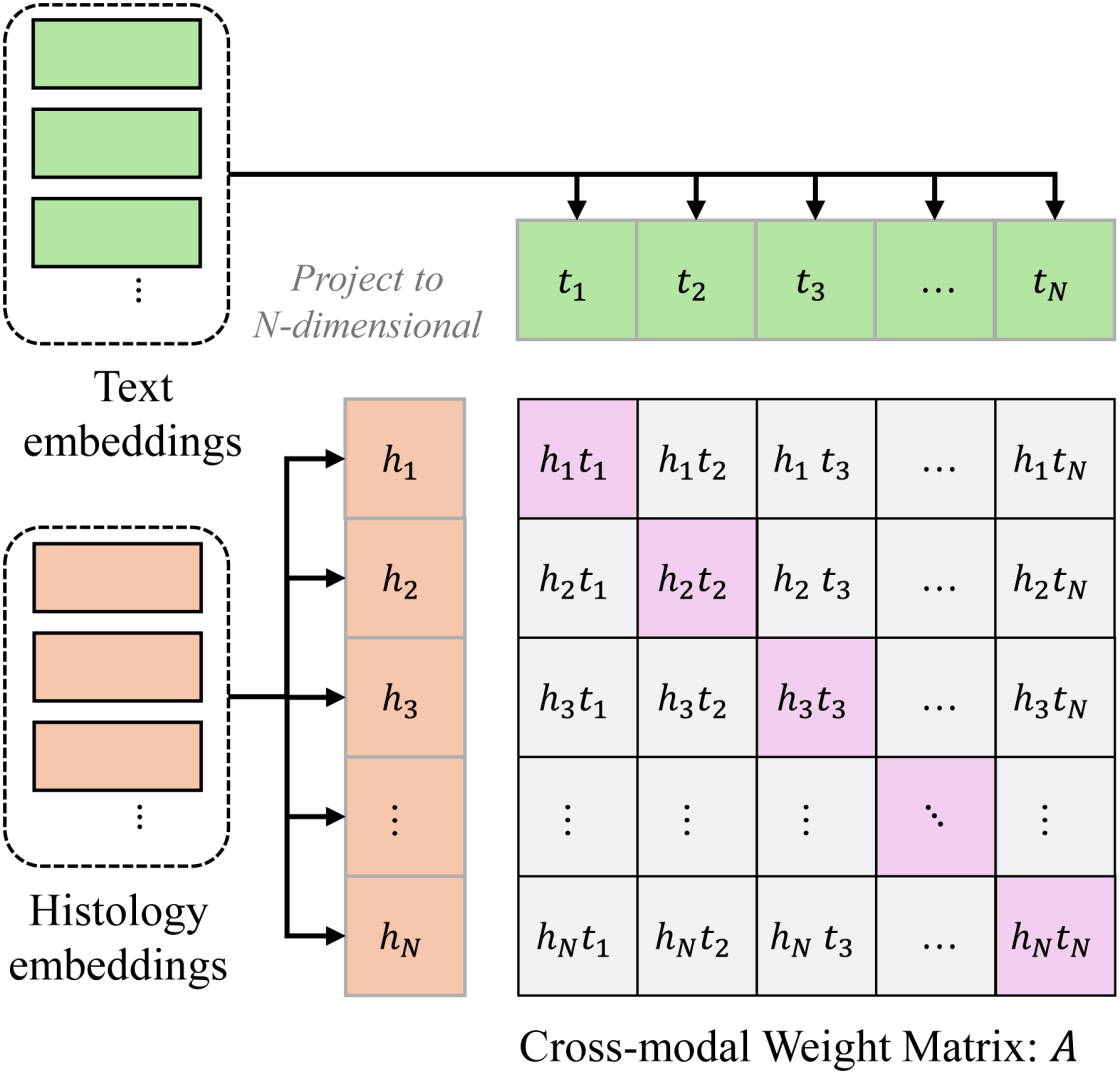
Illustrate a three-modal binding strategy, exemplifying the use of text and histology.

Trimodal binding. In Trimodal Binding, as shown in **Figure 3**, we project both modalities onto the same hidden layer dimension. Subsequently, we employ a method akin to the CLIP (24) to derive a cross-modal weight matrix *A*. However, unlike CLIP, the matrix $A=\text{Pro}(X^{T})⊗\text{Pro}(X^{H})$ obtained in our approach strengthens the interconnection between modalities, rather than being directly utilized for the prediction task. As shown in Equation 5, we use the trimodal binding strategy to transform the modal features into a more closely related feature matrix $X_{T}^{\prime },X_{H}^{\prime },X_{P}^{\prime }$:

(5) $$\begin{array}{ll} X_{T}^{\prime } & =X^{T}\cdot \text{LN}(\text{Pro}(X^{T})⊗\text{Pro}(X^{H}))\\ X_{H}^{\prime } & =X^{H}\cdot \text{LN}(\text{Pro}(X^{H})⊗\text{Pro}(X^{P}))\\ X_{P}^{\prime } & =X^{P}\cdot \text{LN}(\text{Pro}(X^{P})⊗\text{Pro}(X^{T})) \end{array}$$

where *Pro* represents the projection linear layer, ⊗ represents matrix multiplication, *LN* represents layer normalization, $X_{T}^{\prime },X_{H}^{\prime }$ and $X_{P}^{\prime }\in {\mathbb{R}}^{N\times d}$.

#### 3.4.2 Binding Mamba module

**Figure 4:**
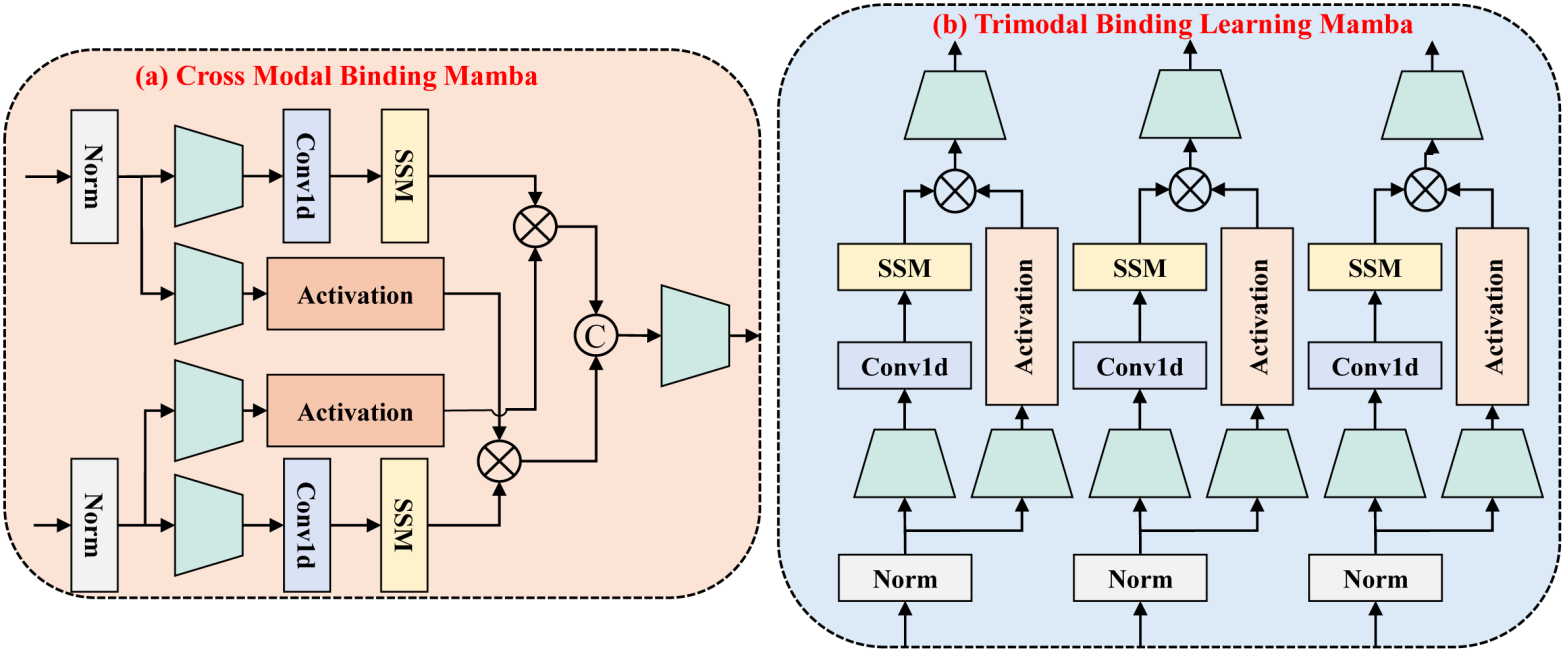
**(a)** Architecture of cross modal binding mamba. **(b)** Architecture of Trimodal Binding Learning Mamba.

To facilitate cross-modal feature interaction and fusion, we design the CrossModal Binding Mamba and the Trimodal Binding Learning Mamba to further encode modality-specific and modality-invariant features, respectively, following the binding learning process. The detailed architectures of these modules are illustrated in **Figure 4**.

Specifically, the Cross-Modal Binding Mamba processes two distinct input features, *X*_1_ and *X*_2_, to produce the fused output representation *X*. This operation is formally defined as follows:

(6) $$X_{1}^{\prime }=\text{SSM}(\text{Conv}1\text{d}(\text{Linear}(\text{LN}(X_{1}))))⊗\text{Act}(\text{Linear}(\text{LN}(X_{2})))$$

(7) $$X_{2}^{\prime }=\text{SSM}(\text{Conv}1\text{d}(\text{Linear}(\text{LN}(X_{2}))))⊗\text{Act}(\text{Linear}(\text{LN}(X_{1})))$$

(8) $$X=\text{Linear}(\text{CAT}(X_{1}^{\prime },X_{2}^{\prime }))$$

The Trimodal Binding Learning Mamba processes three input features, *X*_1_, *X*_2_, and *X*_3_, to produce three corresponding output representations, $X_{1}^{\prime }$, $X_{2}^{\prime }$, and $X_{3}^{\prime }$. This operation is formally defined as follows:

(9) $$\begin{array}{ll} X_{1}^{\prime } & =\text{SSM}(\text{Conv}1\text{d}(\text{Linear}(\text{LN}(X_{1}))))⊗\text{Act}(\text{Linear}(\text{LN}(X_{1})))\\ X_{2}^{\prime } & =\text{SSM}(\text{Conv}1\text{d}(\text{Linear}(\text{LN}(X_{2}))))⊗\text{Act}(\text{Linear}(\text{LN}(X_{2})))\\ X_{3}^{\prime } & =\text{SSM}(\text{Conv}1\text{d}(\text{Linear}(\text{LN}(X_{3}))))⊗\text{Act}(\text{Linear}(\text{LN}(X_{3}))) \end{array}$$

where *Act* represents *SiLU* activation function.

### 3.5 Prediction and optimization

Through the two-stage modeling approach comprising binding learning and Mamba-based backbone encoding, we obtain feature representations enriched with comprehensive cross-modal interactions and task-specific discriminative information. We then employ ABMIL (33) to perform pooling operations on these features. To ensure robust training and effective representation learning, the model is jointly optimized using a combination of three distinct loss functions.

First, the similarity loss (*L*_sim_) facilitates the learning of modality-invariant features by encouraging consistency across different modalities. It is formulated as:

(10) $${ℒ}_{\text{sim}}=\frac{1}{3}\underset{\left(m_{1},m_{2}\right)}{\sum }\{1-\text{sim }(O_{m_{1}}^{I},O_{m_{2}}^{I})\}$$

where sim denotes the cosine similarity function and *O^I^* represents modalityinvariant features.

Second, the difference loss (*L*_diff_) is employed to promote the differentiation of modality-specific features, ensuring that the unique information of each modality is preserved. As defined in Equation 11, it is expressed as:

(11) $${ℒ}_{\text{diff}}=\sum_{m}{\left\|{\left(O_{m}^{I}\right)}^{⊤}O_{m}^{S}\right\|}_{F}^{2}+\sum_{m_{1},m_{2}}{\left\|{\left(O_{m_{1}}^{S}\right)}^{⊤}O_{m_{2}}^{S}\right\|}_{F}^{2}$$

where *O^S^* denotes modality-specific features and ${\left\|\cdot \right\|}_{F}^{2}$ represents the squared Frobenius norm.

Finally, following established paradigms in survival analysis (12–14), we formulate survival analysis as a discrete-time classification problem to effectively handle censored data. The continuous survival timeline is discretized into *n* nonoverlapping intervals. For each patient, the network predicts a hazard vector $H=\left\{h_{1},h_{2},\ldots ,h_{n}\right\}$, where $h_{k}\in \left[0,1\right]$ represents the conditional probability of an event occurring in interval *t_k_*, given the patient survived until the beginning of that interval.

Each patient sample is formalized as a triplet {*H*, *c*, *k*}, where c∈{0,1} is the censorship indicator (*c* = 1 for censored, *c* = 0 for event). The discrete survival function, representing the probability of surviving beyond interval *t_k_*, is defined as:

(12) $$S\left(k\right)=\prod_{i=1}^{k}\left(1-h_{i}\right)$$

The survival prediction objective is optimized via the Negative Log-Likelihood (NLL) loss, which accounts for both observed events and censored instances:

(13) $${ℒ}_{surv}=-c\text{log }(S\left(k\right))-\left(1-c\right)\text{log }(S\left(k-1\right))-\left(1-c\right)\text{log }(h_{k})$$

Hence, the overall loss ${ℒ}_{total}$ of the model is as follows:

(14) $${ℒ}_{total}={ℒ}_{surv}+\lambda_{1}{ℒ}_{sim}+\lambda_{2}{ℒ}_{diff}$$

where *λ*_1_ and *λ*_2_ are weight hyperparameters used to balance the contribution of the auxiliary modality-invariant and modality-specific learning objectives relative to the primary survival analysis task.

## 4 Experiments

### 4.1 Dataset and implementation

Datasets. We evaluated TriBind-Mamba on five diverse cohorts from The Cancer Genome Atlas (TCGA): Breast Invasive Carcinoma (BRCA, *n* = 869), Bladder Urothelial Carcinoma (BLCA, *n* = 359), Stomach Adenocarcinoma (STAD, *n* = 317), Head and Neck Squamous Cell Carcinoma (HNSC, *n* = 392), and Colon and Rectum Adenocarcinoma (COADREAD, *n* = 296).

Survival Outcome. Departing from the common practice of predicting overall survival (OS), we focus on *disease-specific survival* (DSS). This provides a more rigorous assessment of cancer-related mortality by filtering out deaths from unrelated comorbidities, thus ensuring the model captures genuine oncological signals.

Histology Processing. We curated 2,233 diagnostic whole-slide images (WSIs), totaling over 2.86 TB of imaging data. Tissue regions were decomposed into non-overlapping patches of 256 × 256 pixels at 20× magnification (∼0.5 *µ*m/pixel), yielding approximately 32.4 million patches. Morphological features were extracted using the image encoder of CONCH model.

Biological Pathway Tokenization. Transcriptomics data ( $N_{G}=60,499$) and DSS labels were sourced via the Xena platform. To ensure semantic interpretability, we tokenized gene expression levels into biological pathways derived from *Reactome* and *MSigDB-Hallmarks*. Pathway-level transcriptomic tokens represent molecular information as biologically interpretable units corresponding to cellular functions and cancer-related processes, rather than treating thousands of individual genes as independent features. Reactome provides finegrained curated biological processes and signaling pathways, while MSigDBHallmarks summarizes canonical cancer-relevant biological states. Combining these resources allows the transcriptomic modality to cover both detailed pathway-level mechanisms and compact cancer-associated functional programs.

The 90% gene coverage threshold was chosen to ensure that each retained pathway had sufficient measured genes in the TCGA transcriptomic data. If the threshold is too low, more pathways can be included, but many may contain a substantial proportion of unavailable genes, leading to incomplete and noisy pathway representations. If the threshold is too stringent, the retained pathways become highly reliable but many biologically meaningful pathways may be excluded, reducing the diversity of molecular information. Therefore, the 90% threshold was selected as a practical balance between pathway reliability and biological coverage. After applying this fixed filtering rule, we derived a final set of $N_{P}=331$ pathways encompassing 4,999 unique genes. Pathway selection was performed before model training and was independent of survival labels or test-fold performance, thereby avoiding outcome-guided pathway selection.

Experimental Settings. Models were trained using 5-fold cross-validation, with splits stratified by sample site to mitigate batch artifacts. The model was optimized end-to-end for 50 epochs using the AdamW optimizer with a decoupled weight decay of 1 × 10^−5^. We employed a cosine annealing scheduler with an initial learning rate of 1×10^−4^, decaying to a minimum of 1×10^−6^. To handle memory constraints and stabilize training, a patient-level batch size of 1 was used with gradient accumulation (step size = 8). The total objective function ${ℒ}_{total}$integrated discrete-time NLL loss for prognostic tasks, the similarity loss ${ℒ}_{sim}$ (with *λ*_1_ = 0.1) to learn modality-invariant features, and the difference loss ${ℒ}_{\text{diff}}$ (with *λ*_2_ = 0.1) to promote the differentiation of modality-specific features. All experiments were conducted on a high-performance workstation equipped with an NVIDIA GeForce RTX 4090 (24GB VRAM) GPU and an Intel Core i9-13900K CPU.

### 4.2 Comparison with state-of-the-art methods

To demonstrate the superiority of the proposed *TriBind-Mamba*, we conducted a comprehensive comparative analysis against several state-of-the-art (SOTA) methods, categorized by their input modalities. Histology-only Methods: We compared our framework with advanced multiple instance learning (MIL) baselines including ABMIL (33), CLAM (34), and TransMIL (28). Histology & Pathway Methods: This group includes PORPOISE (27), Pathomic Fusion (35), MCAT (14), CMTA (13), and SurvPath (16). Tri-modal Methods: We further compared our model against methods incorporating clinical data, including Metric Learning (36), MultiSurv (37), MMD (38), and HGCN (39).

To further validate the robustness and generalizability of *TriBind-Mamba*, we extended our evaluation by extracting pathological features using two authoritative vision foundation models: UNI (40) and Virchow (41). Since these foundation models are unimodal vision encoders, we utilized the CLIP text encoder to extract semantic features from clinical descriptions, ensuring a unified high-dimensional alignment across all three modalities.

Statistical Reliability of Performance Gains. To further assess whether the observed C-index improvements were statistically reliable, we performed an additional paired bootstrap significance analysis using the out-of-fold patientlevel predictions from the five-fold cross-validation. For each cohort, patients were resampled with replacement, and the C-index difference between TriBindMamba and each competing method was recalculated. This procedure was repeated 1000 times to estimate the 95% confidence interval of the C-index difference and the corresponding bootstrap *p*-value. We focused on representative strong baselines, including SurvPath, MMD, and HGCN, which were the most competitive histology-pathway and tri-modal methods in our experiments. As shown in **Table 1**, TriBind-Mamba consistently achieved positive C-index differences over these baselines, indicating that the performance gain was not only numerically higher but also statistically reliable.

**Table 1:** Bootstrap statistical comparison between TriBind-Mamba and competitive baselines.

Model	Baseline overall C-index	TriBind-Mamba overall C-index	C-index difference	95% CI	Bootstrap *p*-value
SurvPath	0.629	0.664	0.035	[0.018, 0.053]	< 0.001
MMD	0.637	0.664	0.027	[0.012, 0.044]	< 0.001
HGCN	0.639	0.664	0.025	[0.010, 0.041]	< 0.001

According to the experimental results presented in **Tables 2**, **3** and **4**, TriBind-Mamba demonstrates a clear and consistent performance advantage across all five TCGA cohorts. Our detailed analysis reveals several key insights:

**Table 2:** Performance comparison of *TriBind-Mamba* and SOTA methods on five TCGA datasets.

Model/study	BRCA (↑)	BLCA (↑)	STAD (↑)	HNSC (↑)	COADREAD (↑)	Overall (↑)
Histology-only methods						
ABMIL (33)	0.493 ± 0.126	0.518 ± 0.078	0.550 ± 0.077	0.580 ± 0.019	0.630 ± 0.102	0.554
CLAM (34)	0.512 ± 0.084	0.536 ± 0.065	0.562 ± 0.071	0.545 ± 0.052	0.612 ± 0.098	0.553
TransMIL (28)	0.530 ± 0.057	0.551 ± 0.091	0.544 ± 0.080	0.523 ± 0.043	0.632 ± 0.143	0.556
Histology & pathway methods						
PORPOISE (27)	0.582 ± 0.091	0.574 ± 0.063	0.568 ± 0.102	0.556 ± 0.074	0.598 ± 0.115	0.576
Pathomic Fusion (35)	0.615 ± 0.083	0.566 ± 0.038	0.525 ± 0.140	0.566 ± 0.066	0.584 ± 0.109	0.571
MCAT (14)	0.652 ± 0.117	0.598 ± 0.094	0.557 ± 0.101	0.531 ± 0.049	0.634 ± 0.204	0.594
CMTA (13)	0.648 ± 0.082	0.612 ± 0.075	0.583 ± 0.066	0.589 ± 0.054	0.665 ± 0.152	0.619
SurvPath (16)	0.655 ± 0.089	0.625 ± 0.056	0.592 ± 0.047	0.600 ± 0.061	0.673 ± 0.170	0.629
Tri-modal methods (histology + pathway + clinical)						
Metric Learning (36)	0.631 ± 0.095	0.608 ± 0.082	0.571 ± 0.093	0.576 ± 0.068	0.645 ± 0.182	0.606
MultiSurv (37)	0.642 ± 0.104	0.615 ± 0.071	0.588 ± 0.081	0.592 ± 0.073	0.651 ± 0.134	0.618
MMD (38)	0.661 ± 0.075	0.638 ± 0.064	0.614 ± 0.058	0.605 ± 0.049	0.668 ± 0.121	0.637
HGCN (39)	0.668 ± 0.083	0.634 ± 0.059	0.609 ± 0.072	0.612 ± 0.055	0.662 ± 0.144	0.639
**TriBind-Mamba (Ours)**	**0.689 ± 0.074**	**0.658 ± 0.052**	**0.632 ± 0.046**	**0.641 ± 0.058**	**0.702 ± 0.118**	**0.664**

Results are reported as C-index (mean ± std). The best performance is in bold, and the second best is underlined.

**Table 3:** Performance comparison using UNI (40) extracted features on five TCGA datasets.

Model/Study	BRCA (↑)	BLCA (↑)	STAD (↑)	HNSC (↑)	COADREAD (↑)	Overall (↑)
Histology-only methods						
ABMIL (33)	0.491 ± 0.124	0.516 ± 0.075	0.548 ± 0.079	0.578 ± 0.021	0.628 ± 0.104	0.552
CLAM (34)	0.510 ± 0.082	0.534 ± 0.067	0.560 ± 0.073	0.543 ± 0.054	0.610 ± 0.101	0.551
TransMIL (28)	0.528 ± 0.059	0.549 ± 0.093	0.542 ± 0.082	0.521 ± 0.045	0.630 ± 0.141	0.554
Histology & pathway methods						
PORPOISE (27)	0.580 ± 0.093	0.572 ± 0.061	0.566 ± 0.104	0.554 ± 0.072	0.596 ± 0.113	0.574
Pathomic Fusion (35)	0.613 ± 0.085	0.564 ± 0.036	0.523 ± 0.142	0.564 ± 0.064	0.582 ± 0.107	0.569
MCAT (14)	0.650 ± 0.115	0.596 ± 0.092	0.555 ± 0.103	0.529 ± 0.047	0.632 ± 0.202	0.592
CMTA (13)	0.646 ± 0.084	0.610 ± 0.073	0.581 ± 0.064	0.587 ± 0.052	0.663 ± 0.154	0.617
SurvPath (16)	0.653 ± 0.091	0.623 ± 0.054	0.590 ± 0.045	0.598 ± 0.063	0.671 ± 0.172	0.627
Tri-modal methods (histology + pathway + clinical)						
Metric Learning (36)	0.629 ± 0.093	0.606 ± 0.084	0.569 ± 0.091	0.574 ± 0.066	0.643 ± 0.180	0.604
MultiSurv (37)	0.640 ± 0.102	0.613 ± 0.073	0.586 ± 0.079	0.590 ± 0.075	0.649 ± 0.132	0.616
MMD (38)	0.659 ± 0.077	0.636 ± 0.062	0.612 ± 0.056	0.603 ± 0.047	0.666 ± 0.123	0.635
HGCN (39)	0.666 ± 0.081	0.632 ± 0.057	0.607 ± 0.074	0.610 ± 0.053	0.660 ± 0.142	0.637
**TriBind-Mamba (Ours)**	**0.687 ± 0.076**	**0.656 ± 0.054**	**0.630 ± 0.048**	**0.639 ± 0.056**	**0.700 ± 0.116**	**0.662**

Results are reported as C-index (mean ± std). The best performance is in bold, and the second best is underlined.

**Table 4:** Performance comparison using Virchow (41) extracted features on five TCGA datasets.

Model/Study	BRCA (↑)	BLCA (↑)	STAD (↑)	HNSC (↑)	COADREAD (↑)	Overall (↑)
Histology-only methods						
ABMIL (33)	0.495 ± 0.128	0.520 ± 0.080	0.552 ± 0.075	0.582 ± 0.017	0.632 ± 0.100	0.556
CLAM (34)	0.514 ± 0.086	0.538 ± 0.063	0.564 ± 0.069	0.547 ± 0.050	0.614 ± 0.096	0.555
TransMIL (28)	0.532 ± 0.055	0.553 ± 0.089	0.546 ± 0.078	0.525 ± 0.041	0.634 ± 0.145	0.558
Histology & pathway methods						
PORPOISE (27)	0.584 ± 0.089	0.576 ± 0.065	0.570 ± 0.100	0.558 ± 0.076	0.600 ± 0.117	0.578
Pathomic Fusion (35)	0.617 ± 0.081	0.568 ± 0.040	0.527 ± 0.138	0.568 ± 0.068	0.586 ± 0.111	0.573
MCAT (14)	0.654 ± 0.119	0.600 ± 0.096	0.559 ± 0.099	0.533 ± 0.051	0.636 ± 0.206	0.596
CMTA (13)	0.650 ± 0.080	0.614 ± 0.077	0.585 ± 0.068	0.591 ± 0.056	0.667 ± 0.150	0.621
SurvPath (16)	0.657 ± 0.087	0.627 ± 0.058	0.594 ± 0.049	0.602 ± 0.059	0.675 ± 0.168	0.631
Tri-modal methods (histology + pathway + clinical)						
Metric Learning (36)	0.633 ± 0.097	0.610 ± 0.080	0.573 ± 0.095	0.578 ± 0.070	0.647 ± 0.184	0.608
MultiSurv (37)	0.644 ± 0.106	0.617 ± 0.069	0.590 ± 0.083	0.594 ± 0.071	0.653 ± 0.136	0.620
MMD (38)	0.663 ± 0.073	0.640 ± 0.066	0.616 ± 0.060	0.607 ± 0.051	0.670 ± 0.119	0.639
HGCN (39)	0.670 ± 0.085	0.636 ± 0.061	0.611 ± 0.070	0.614 ± 0.057	0.664 ± 0.146	0.641
**TriBind-Mamba (Ours)**	**0.691 ± 0.072**	**0.660 ± 0.050**	**0.634 ± 0.044**	**0.643 ± 0.060**	**0.704 ± 0.120**	**0.666**

Results are reported as C-index (mean ± std). The best performance is in bold, and the second best is underlined.

Superiority of Tri-modal Integration. Our proposed framework significantly outperforms histology-only and bimodal histology-pathway models. As shown in **Table 2**, TriBind-Mamba achieves an overall C-index of 0.664, which is a substantial improvement over the best histology-only baseline (TransMIL, 0.556) and the leading bimodal baseline (SurvPath, 0.629). This performance gap underscores the importance of clinical prompts in guiding the fusion process and providing a more comprehensive understanding of patient prognosis.

Robustness Across Foundation Models. One of the most critical findings is the stability of our model when utilizing different pathological foundation models. As observed in **Tables 3** and **4**, TriBind-Mamba consistently maintains its state-of-the-art (SOTA) position, regardless of whether UNI or Virchow features are employed. While the overall performance levels remain comparable across different backbones, our model effectively leverages the high-quality representations from these foundation models to achieve stable results (e.g., reaching a C-index of 0.704 on COADREAD with Virchow).

Generalizability Across Diverse Cancer Types. TriBind-Mamba achieves the best performance (bold) in all tested datasets across all three feature extraction schemes. Notably, in highly heterogeneous cancers such as STAD, our model consistently outperforms SurvPath by approximately 4.0% in C-index. This consistent gain across different organs and tissue types highlights the framework’s ability to adapt to varying morphological and biological contexts.

Advantages of the Mamba Architecture. Unlike Transformer-based models such as MCAT or SurvPath, which suffer from quadratic complexity with respect to the number of patches, our State Space Model (SSM) based architecture enables the modeling of much denser cross-modal interactions. By avoiding the computational bottlenecks of global self-attention, TriBind-Mamba is able to more effectively correlate thousands of histology patches with highdimensional pathway and clinical tokens, leading to superior predictive accuracy and more refined multimodal semantic alignment.

### 4.3 Efficiency analysis

To further evaluate the computational advantages of the proposed TriBindMamba framework, we conducted a comprehensive evaluation of its efficiency. Beyond predictive performance, we assessed the computational efficiency of our model across multiple metrics: the number of trainable parameters (Param.), floating-point operations (FLOPs), single-sample inference time (Time), and peak memory usage (Mem.). The comparative results against representative state-of-the-art (SOTA) survival models are summarized in the efficiency comparison table.

**Table 5:** Efficiency comparison of TriBind-Mamba and representative baselines. FLOPs, Time, and Memory are computed using a representative WSI sample with 4,096 patches as input.

Model	Param. (M)	FLOPs (G)	Time (S)	Mem. (G)	Overall C-index
ABMIL 33	0.45	8.2	0.012	1.05	0.554
TransMIL 28	2.68	142.5	0.185	8.42	0.556
MCAT (14)	1.85	110.2	0.142	6.50	0.594
SurvPath 16	2.14	165.8	0.210	9.85	0.629
HGCN 39	3.12	85.4	0.115	4.22	0.639
TriBind-Mamba (Ours)	1.87	92.4	0.028	3.15	0.664

The experimental results demonstrate that TriBind-Mamba achieves an optimal balance between effectiveness and efficiency by maintaining a significantly lower computational footprint of 92.4G FLOPs and 3.15G Memory while reaching the highest Overall C-index of 0.664 compared to lightweight histology-only methods and memory-intensive Transformer-based models like SurvPath. A critical advantage of the Mamba-based architecture is its linear complexity relative to sequence length, which enables the framework to process tri-modal inputs in only 0.028 seconds, making it approximately 7.5 times faster than SurvPath.

By avoiding the quadratic memory bottlenecks inherent in traditional Transformers, our model remains parameter-efficient at 1.87M despite incorporating Clinical Knowledge Prompting as an additional modality. This streamlined design allows the model to capture global synergistic interactions across clinical priors, morphological features, and genomic profiles without redundant training overhead, rendering it highly suitable for real-time clinical decision support systems where resource conservation and low-latency assessment are critical.

The GPT-4-based clinical narrative generation is performed only once as an offline preprocessing step. The generated narratives are cached and reused during all subsequent training, validation, and testing procedures. Therefore, GPT-4 does not increase the per-epoch training cost or the final model inference cost. After preprocessing, TriBind-Mamba only takes fixed clinical text embeddings as input. Moreover, as shown in Section 4.4.4, rule-based clinical narratives and open-source LLM-generated narratives also provide performance gains over raw structured clinical variables, indicating that the CKP module remains reproducible and scalable without requiring proprietary API access.

### 4.4 Ablation study

To thoroughly evaluate the contribution of each component within the *TriBindMamba* framework, we conducted a series of ablation experiments on five TCGA cohorts. The results, categorized into four distinct perspectives, demonstrate the necessity of tri-modal integration, the efficiency of the Mamba-based backbone, the effectiveness of our joint optimization strategy, and the added value of standardized clinical knowledge prompting.

#### 4.4.1 Effectiveness of modality integration

We first assessed the impact of individual modalities to determine if the combination of histology, pathways, and clinical prompts provides complementary prognostic signals. As shown in **Table 5**, removing the clinical knowledge prompting leads to a significant performance drop, with the overall C-index decreasing from 0.664 to 0.627. This suggests that high-level semantic priors generated by LLMs provide critical prognostic anchors that histology and transcriptomics alone cannot capture. Similarly, excluding biological pathways (Histology + Clinical) results in a decrease to 0.631, highlighting that transcriptomic profiles offer essential molecular-level insights into cellular functions that are complementary to morphological features.

**Table 5:** Ablation study on modality integration across five TCGA datasets. Results are reported as C-index.

Modality combinations	BRCA	BLCA	STAD	HNSC	COADREAD	Overall
Histology Only	0.530	0.551	0.544	0.523	0.632	0.556
Histology + Pathway	0.655	0.625	0.592	0.600	0.673	0.627
Histology + Clinical	0.658	0.628	0.598	0.604	0.678	0.631
TriBind-Mamba (Full)	0.689	0.658	0.632	0.641	0.702	0.664

#### 4.4.2 Role of modality binding and Mamba architecture

Next, we evaluated the impact of our fine-grained design, specifically the binding mechanism and the Mamba-based backbone. To verify the efficacy of our proposed Binding Learning strategy, we replaced it with a simple concatenationbased fusion. The performance dropped to 0.618 (**Table 6**), proving that our Bimodal and Trimodal Binding strategies effectively facilitate deeper cross-modal alignment by projecting features into a shared semantic space. Furthermore, we conducted an experiment replacing the Mamba blocks with standard MultiHead Self-Attention (MHSA) layers. The Transformer-based variant achieved a lower C-index (0.621) and exhibited significantly higher memory consumption when processing long patch sequences (*N_H_ >* 50,000), where the quadratic complexity of attention becomes a bottleneck. The linear complexity of Mamba allows our model to capture long-range dependencies across ultra-long tri-modal sequences more efficiently.

**Table 6:** Evaluation of modality binding strategies and backbone architectures.

Architectural components	BRCA	BLCA	STAD	HNSC	COADREAD	Overall
w/o Binding Learning (Concat)	0.641	0.615	0.584	0.592	0.660	0.618
w/o Mamba (Transformer)	0.645	0.619	0.589	0.595	0.665	0.621
**TriBind-Mamba (Full)**	**0.689**	**0.658**	**0.632**	**0.641**	**0.702**	**0.664**

Bold values indicate the best performance in each column.

#### 4.4.3 Role of joint optimization strategy and loss-weight sensitivity analysis

Finally, we investigated the influence of our designed auxiliary loss functions and joint learning strategy. We quantified the extent to which the model learns modality-invariant and modality-specific representations by removing ${ℒ}_{sim}$ and ${ℒ}_{\text{diff}}$. As shown in **Table 7**, training the model solely with the survival loss ( ${ℒ}_{surv}$) led to a degraded overall C-index of 0.604. This drop confirms that explicitly distinguishing between modality-invariant and modality-specific features helps prevent “modality collapse” and ensures that the model does not over-rely on a single dominant modality, thereby enhancing overall robustness. The similarity loss aids in capturing shared biological signals across phenotypes and genotypes, while the difference loss helps the model capture fine-grained distinctions between morphological patterns, molecular pathways, and clinical records.

**Table 7:** Comparison of different joint loss function combinations.

Learning strategy	BRCA	BLCA	STAD	HNSC	COADREAD	Overall
w/o ${ℒ}_{sim}$ (Similarity Loss)	0.635	0.610	0.578	0.589	0.655	0.613
w/o ${ℒ}_{diff}$ (Difference Loss)	0.628	0.605	0.575	0.585	0.648	0.608
w/o ${ℒ}_{sim}$ & ${ℒ}_{diff}$ (Task Only)	0.624	0.602	0.572	0.581	0.642	0.604
**TriBind-Mamba (Full)**	**0.689**	**0.658**	**0.632**	**0.641**	**0.702**	**0.664**

Bold values indicate the best performance in each column.

**Figure 5:**
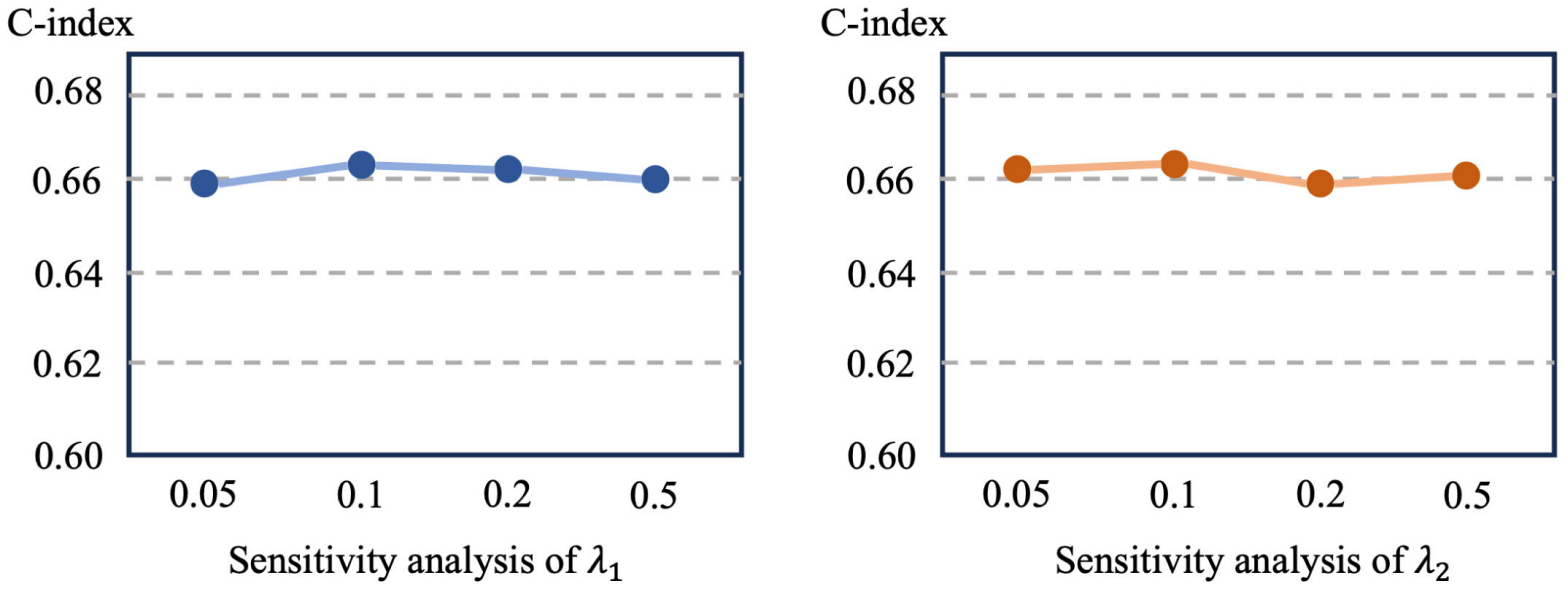
Sensitivity analysis of *λ*_1_ and *λ*_2_. The results are obtained by varying the value of *λ*_1_ and *λ*_2_, while fixing the other hyperparameters to the values adopted in the experiments.

To further evaluate the stability of the joint optimization strategy, we conducted a loss-weight sensitivity analysis for *λ*_1_ and *λ*_2_, which control the contributions of ${ℒ}_{sim}$ and ${ℒ}_{\text{diff}}$, respectively. Specifically, we varied each coefficient within {0.05,0.1,0.2,0.5} while keeping the other coefficient fixed at 0.1, and evaluated the resulting overall C-index. As shown in **Figure 5**, the performance remained relatively stable across this range for both coefficients, indicating that the model is not overly sensitive to small changes in the auxiliary loss weights. The best or near-best performance was achieved when both *λ*_1_ and *λ*_2_ were set to 0.1, which provides a balanced contribution between the primary survival prediction objective and the auxiliary multimodal representation learning objectives.

#### 4.4.4 Clinical knowledge prompting sensitivity analysis

Although the modality ablation in **Table 5** demonstrates that clinical knowledge prompting contributes to survival prediction, it does not isolate whether the improvement is specifically attributable to GPT-4-generated narratives or to the inclusion of standardized clinical information more generally. To address this issue, we conducted a Clinical Knowledge Prompting sensitivity analysis by comparing different clinical information encoding strategies under identical data splits, model architecture, and optimization settings.

We evaluated five variants. First, the “Histology + Pathway” setting removes the clinical modality entirely and serves as the non-clinical baseline. Second, the “Raw structured clinical variables” setting directly encodes normalized continuous variables and one-hot categorical clinical variables using a lightweight multilayer perceptron, without converting them into natural language. Third, the “Rule-based clinical narratives” setting converts the same structured clinical variables into fixed natural language sentences using a deterministic template. Fourth, the “Open-source LLM narratives” setting uses Qwen2.5-7B-Instruct with the same fixed prompt template as GPT-4. Finally, the “GPT-4 clinical narratives” setting corresponds to the default TriBind-Mamba implementation.

As shown in **Table 8**, incorporating clinical information consistently improved survival prediction over the non-clinical baseline, with the overall C-index increasing from 0.627 to 0.642–0.664. Narrative-based CKP outperformed direct encoding of raw structured variables, indicating the benefit of transforming tabular clinical records into standardized semantic text. Importantly, LLMgenerated narratives further outperformed rule-based narratives, with opensource LLM and GPT-4 variants achieving overall C-index values of 0.658 and 0.664, respectively, compared with 0.651 for the rule-based template. This suggests that LLM-generated descriptions provide additional semantic value beyond deterministic template generation, likely by producing more natural, coherent, and contextually normalized clinical narratives. Nevertheless, the competitive performance of the rule-based variant indicates that TriBind-Mamba remains reproducible without proprietary API access, while GPT-4 provides the strongest performance through enhanced semantic normalization.

**Table 8:** Clinical knowledge Prompting sensitivity analysis across five TCGA cohorts.

CKP encoding strategy	BRCA	BLCA	STAD	HNSC	COADREAD	Overall
w/o clinical modality	0.655	0.625	0.592	0.600	0.673	0.627
Raw structured clinical variables	0.667	0.636	0.608	0.615	0.685	0.642
Rule-based clinical narratives	0.676	0.645	0.619	0.626	0.691	0.651
Open-source LLM-generated narratives	0.682	0.651	0.625	0.634	0.696	0.658
**GPT-4-generated clinical narratives**	**0.689**	**0.658**	**0.632**	**0.641**	**0.702**	**0.664**

Bold values indicate the best performance in each column.

### 4.5 Interpretability

#### 4.5.1 Patient risk stratification and survival analysis

To demonstrate the clinical utility and prognostic power of the *TriBindMamba* framework, we conducted a comprehensive risk stratification analysis using Kaplan-Meier (KM) survival curves and log-rank tests across all five TCGA cohorts. This analysis serves to validate whether the continuous risk scores generated by our tri-modal model can be effectively translated into statistically significant clinical strata.

For each cohort, patients were stratified into high-risk (red) and low-risk (blue) groups based on the median value of the predicted risk scores. The survival trajectories of these groups were then estimated using the KaplanMeier method, and the statistical significance of the separation between the groups was assessed using the log-rank test, with a significance threshold of *P<* 0.05.

**Figure 6:**
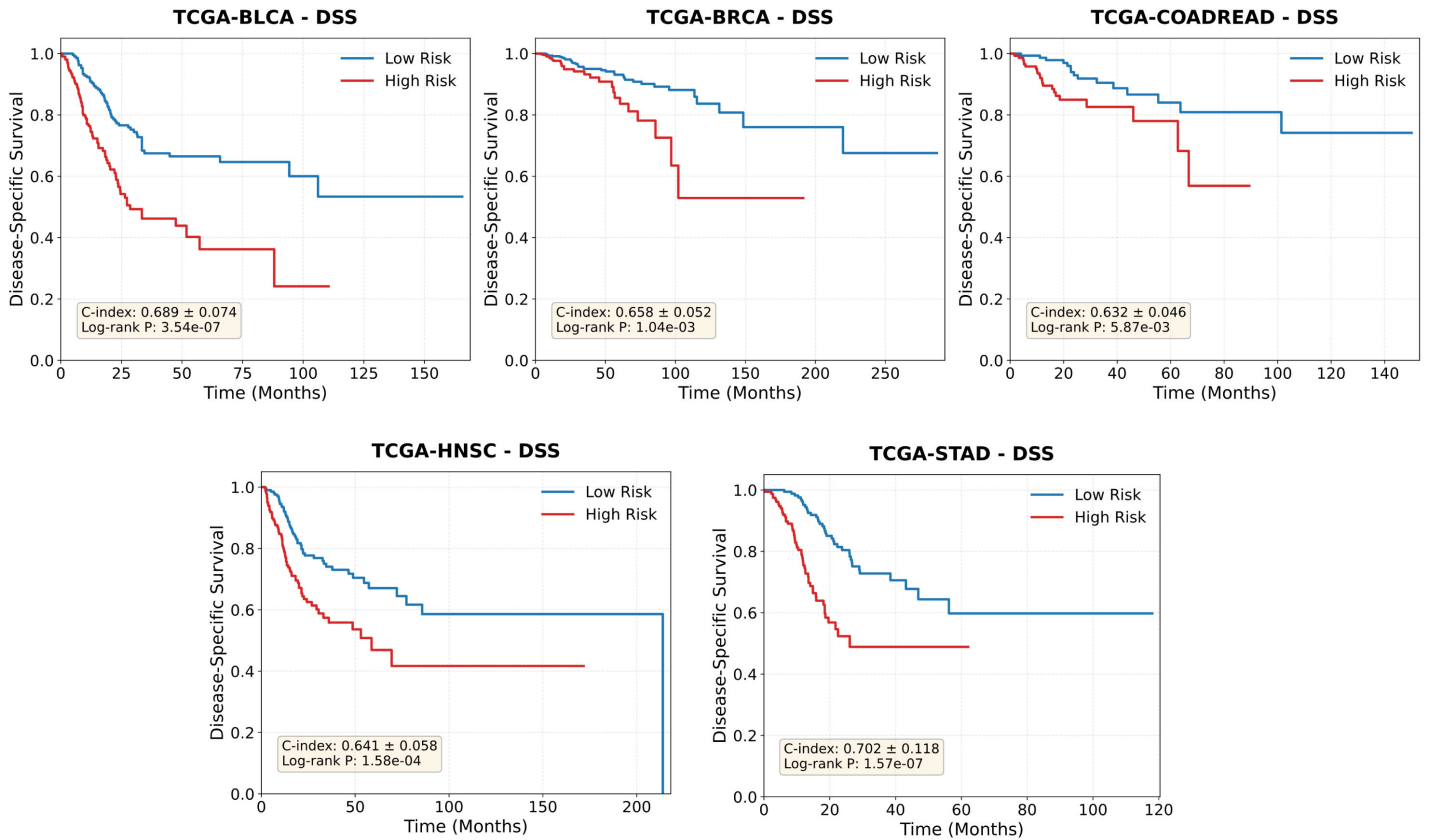
Kaplan-Meier survival curves for Disease-Specific Survival (DSS) stratification on five TCGA cohorts. The p-values obtained via log-rank tests demonstrate the statistically significant (*P<* 0.05) discriminative capability of the TriBind-Mamba model.

As illustrated in **Figure 6**, our framework achieved a clear and robust separation between the risk groups across BRCA, BLCA, STAD, HNSC, and COADREAD datasets. The consistently small p-values (*P<* 0.05) confirm that the tri-modal integration of histology, pathways, and clinical prompts successfully captures essential prognostic signals that differentiate patient outcomes. The wide margin between the high-risk and low-risk curves indicates that TriBindMamba can reliably identify patients with poor prognosis, thereby providing valuable decision support for clinicians in determining patient stratification and personalized therapeutic strategies.

#### 4.5.2 Interpretation analysis through WSI heatmap visualizations

To elucidate the decision-making process of TriBind-Mamba, we conducted a multi-level interpretability analysis by projecting attention scores back onto the original Whole Slide Images (WSIs). This visualization provides a modelderived view of the association between the model’s high-dimensional reasoning and observable histopathological phenotypes.

**Figure 7:**
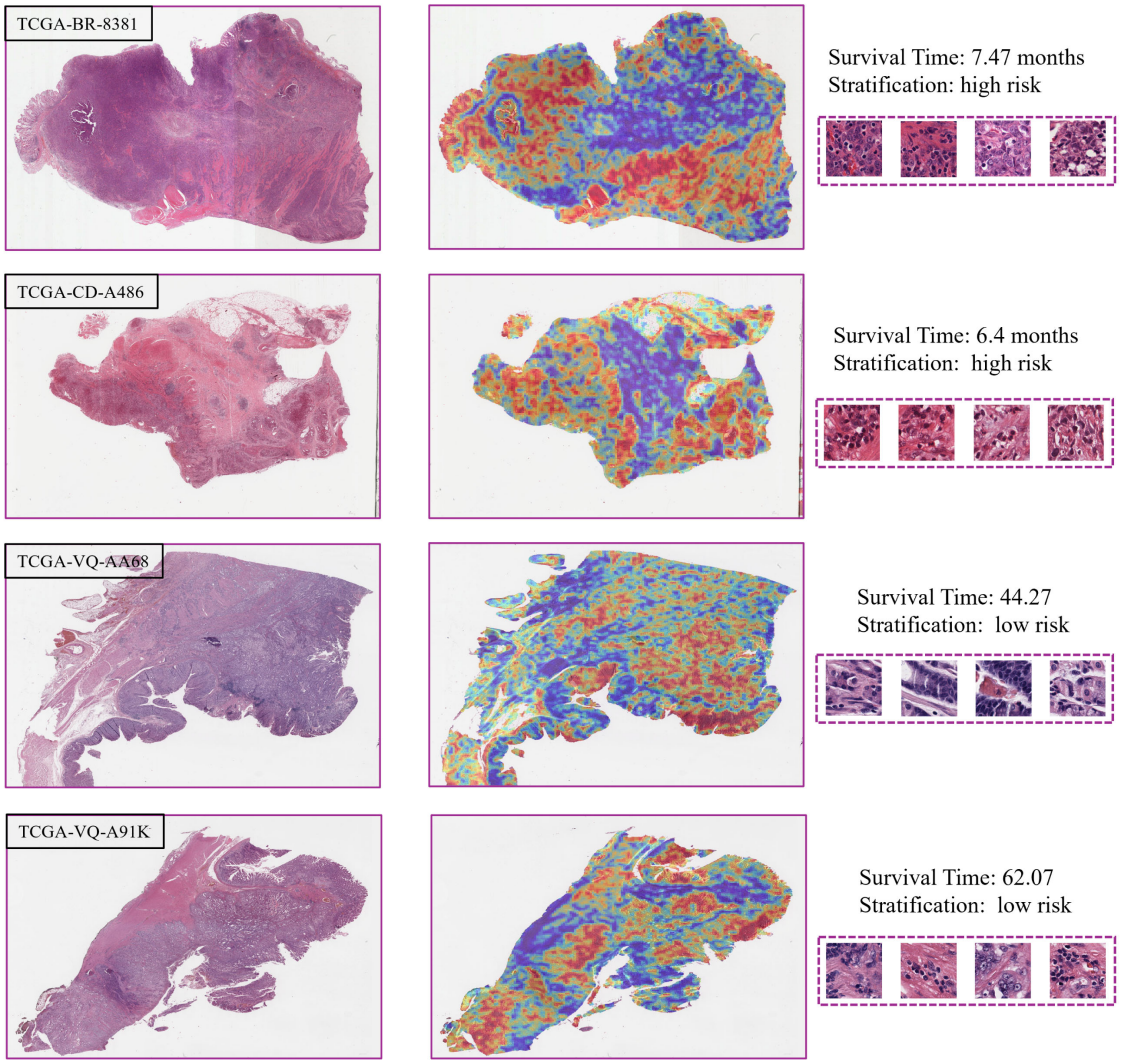
Interpretation analysis via WSI heatmap visualizations.

As illustrated in **Figure 7**, the generated heatmaps utilize a color-coded importance scale, where red (hot) regions indicate high-attention areas that heavily influence the survival risk assessment, and blue (cold) regions signify areas of lower prognostic contribution. At the macro-level, the model tends to localize tumor-associated regions, specifically focusing on the invasive front and cellulardense areas, which is consistent with regions commonly considered informative in routine diagnostic pathology.

At the micro-level, we visualized the four image patches with the highest attention weights from high-risk patients to analyze specific morphological patterns associated with prognosis. These high-attention regions exhibit characteristic malignant features, including marked nuclear pleomorphism, hyperchromasia, and irregular nuclear contours. Furthermore, the presence of architectural distortion, increased nuclear-to-cytoplasmic ratios, and tumor cell invasion into the stroma within these regions suggests that the framework captures aggressive morphological signatures associated with poor Disease-Specific Survival (DSS).

It should be noted that the TCGA WSIs used in this study do not provide board-certified pathologist annotations of regions of interest, such as tumor nests, invasive margins, or stromal invasion areas. Therefore, the current heatmap analysis should be interpreted as a qualitative model-derived explanation rather than formal pathological ground truth. Future work will further validate the attention maps using expert-annotated regions of interest from independent clinical cohorts.

#### 4.5.3 Interpretation analysis of pathways contribution

To further improve the biological interpretability of TriBind-Mamba, we analyzed the contribution of pathway-level transcriptomic tokens to survival risk prediction across the five cancer cohorts. Specifically, for each patient, we computed the Integrated Gradients (IG) score of each pathway token with respect to the predicted survival risk. The absolute IG scores were then averaged across all patients and cross-validation folds within each cohort to obtain a cohort-level pathway importance ranking.

**Figure 8:**
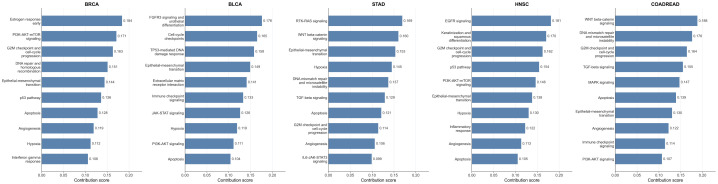
Interpretation analysis of pathway contribution. For each cohort, the top 10 pathway-level transcriptomic tokens are ranked by average absolute Integrated Gradients contribution score across patients and cross-validation folds.

As shown in **Figure 8**, the horizontal bar plots summarize the dominant pathway-level signals contributing to survival risk prediction. The identified pathways reveal both shared pan-cancer programs and cohort-specific molecular patterns. Several recurrent pathways, including epithelial-mesenchymal transition, G2M checkpoint and cell-cycle progression, DNA repair, apoptosis, hypoxia, angiogenesis, PI3K-AKT-mTOR signaling, and immune checkpoint signaling, appeared among the top contributors across multiple cohorts. These processes are closely associated with tumor aggressiveness, proliferative activity, genomic instability, tumor-stroma interaction, immune regulation, and poor clinical outcome, suggesting that the pathway branch captures biologically meaningful prognostic signals.

Representative cohort-specific patterns were also observed. In BRCA, estrogen response, PI3K-AKT-mTOR signaling, DNA repair, and epithelial-mesenchymal transition were among the major contributors, reflecting hormone-related signaling, proliferative activity, and tumor progression. In HNSC, EGFR signaling, keratinization and squamous differentiation, p53 pathway, PI3K-AKT-mTOR signaling, and inflammatory response contributed strongly, highlighting molecular mechanisms relevant to squamous tumor progression, epithelial differentiation, and tumor-associated inflammation. These findings indicate that TriBindMamba captures both common oncogenic programs and disease-relevant molecular heterogeneity across cancer types.

## 5 Discussion

The experimental results and interpretability analyses presented in this study underscore the efficacy of TriBind-Mamba as a robust framework for multimodal survival prediction. By successfully integrating clinical priors, histological morphology, and biological pathways through a Mamba-based architecture, our model addresses several long-standing challenges in computational pathology.

### 5.1 Synergy of tri-modal learning and clinical prompting

A primary finding of this work is the significant performance gain afforded by Clinical Knowledge Prompting (CKP). Most existing multimodal models, such as SurvPath and MCAT, rely primarily on the interaction between imaging and genomics. However, our results demonstrate that discrete clinical indicators (e.g., stage, grade, age), when expanded into semantic narratives via LLMs, provide high-level prognostic anchors that guide the alignment of low-level morphological and molecular features. This tri-modal integration effectively mitigates the informational sparsity inherent in single modalities and prevents “modality collapse” where the model over-relies on a single dominant source of data. The similarity and dissimilarity losses further ensure that the model captures both shared biological signals and unique modality-specific nuances, leading to superior robustness across diverse cancer cohorts.

Across cancers including HNSC, CKP may improve the alignment between clinical priors and disease-relevant pathological or molecular patterns. Clinical variables such as tumor stage, T/N/M category, age, and histological grade reflect tumor burden, local invasion, nodal spread, metastatic potential, and disease aggressiveness. When represented as standardized clinical narratives, these priors may guide the model to better associate high-risk clinical context with histological patterns such as tumor-dense regions, invasive growth, tumorstroma interfaces, nuclear pleomorphism, and stromal invasion, as well as transcriptomic programs related to proliferation, epithelial-mesenchymal transition, extracellular matrix remodeling, hypoxia, and immune regulation. In HNSC, these associations are particularly relevant because local invasion, nodal involvement, tumor-stroma interactions, epithelial differentiation, and inflammatory responses are central to clinical risk stratification and disease progression. Thus, CKP may provide a clinically meaningful semantic anchor for integrating cancer-relevant morphology and pathway-level molecular information. This interpretation is biologically plausible but remains hypothesis-generating, and future work with cancer-specific expert annotations and external clinical cohorts will be needed for further validation.

### 5.2 Clinical utility and interpretability

Beyond numerical performance, the clinical utility of our framework is supported through risk stratification and visualization. The wide separation in Kaplan-Meier curves across all five TCGA datasets indicates that the modelpredicted risk scores translate into statistically significant clinical strata, enabling the identification of high-risk patients who may benefit from closer monitoring or more aggressive interventions. Furthermore, the attention heatmaps and pathway contribution analyses suggest that TriBind-Mamba focuses on tumor-dense regions, aggressive morphologies, and biologically meaningful molecular programs, such as nuclear pleomorphism, stromal invasion, epithelial-mesenchymal transition, hypoxia, angiogenesis, and immune regulation. These complementary interpretability analyses provide useful model-derived explanations while avoiding the assumption that attention weights alone constitute pathological ground truth.

### 5.3 Limitations and future work

Despite the promising results, this study has several limitations. First, although TriBind-Mamba was validated on five heterogeneous TCGA cohorts, future studies should further evaluate its generalizability on independent institutional cohorts. We are currently collecting non-TCGA multimodal survival cohorts with matched WSIs, transcriptomic profiles, clinical records, and longterm follow-up data. Second, this study mainly focuses on risk ranking and patient stratification using C-index and Kaplan-Meier analysis, following common practice in multimodal computational pathology survival prediction. Future work may further assess calibration plots, Brier score, and time-dependent AUC in external cohorts with standardized follow-up and clinically defined prediction horizons. Third, although the heatmap and pathway contribution analyses provide useful qualitative interpretability, expert-annotated regions of interest are unavailable in TCGA WSIs. Future work will incorporate pathologist annotations and may extend TriBind-Mamba to Cox-based or continuoustime survival modeling. Finally, the current experiments focus on the standard complete-modality benchmark setting. Real-world deployment will require dedicated missing-modality and noisy-modality strategies, such as modality dropout, modality masking, cross-modal reconstruction, uncertainty-aware fusion, and robust clinical text encoding.

## 6 Conclusion

In this paper, we introduced TriBind-Mamba, a novel tri-modal binding learning framework designed for cancer survival prediction. By leveraging Clinical Knowledge Prompting and a Mamba-based linear-time architecture, we have demonstrated that it is possible to achieve state-of-the-art prognostic accuracy while significantly reducing the computational burden associated with long-sequence modeling in digital pathology.

Our empirical evidence across five diverse TCGA cohorts confirms that the synergy between clinical narratives, histological patterns, and biological pathways provides a more holistic and accurate representation of patient prognosis than unimodal or bimodal approaches. Furthermore, our joint optimization strategy ensures stable representation learning, while our interpretability framework bridges the gap between high-dimensional features and observable pathological phenotypes. We believe that TriBind-Mamba represents a significant step forward in developing efficient, interpretable, and clinically relevant multimodal AI systems for precision medicine, offering a powerful tool for personalized patient management and survival analysis.

## Data Availability

The original contributions presented in the study are included in the article/supplementary material. Further inquiries can be directed to the corresponding author.

## Appendix

### Appendix 1 Prompt template and generation settings for clinical knowledge prompting

The following fixed prompt template was used for GPT-4 and open-source LLM-based clinical narrative generation:

Generation settings were kept fixed across all patients and cohorts: the same prompt template, input fields, missing-value handling rule, and single-paragraph output constraint were applied to every clinical record. Clinical narrative generation was performed once as an offline preprocessing step before cross-validation, and the resulting narratives were cached and reused unchanged during model training, validation, and testing. No survival time, censoring status, recurrence, treatment response, mortality, risk group, or fold-specific information was included in the prompt input.

You are a clinical oncology assistant. Convert the following structured clinical variables into one concise and neutral clinical narrative for multimodal survival prediction.

Rules:

1. Use only the information explicitly provided in the structured variables.
2. Do not infer survival outcome, prognosis, treatment response, recurrence, mortality, or risk group.
3. Do not add any clinical facts that are not present in the input.
4. If a variable is missing, state that it is unavailable.
5. Keep the output factual, standardized, and suitable for text encoding.

Input variables: Cancer type: {cancer_type}; Age at diagnosis: {age}; Sex: {sex}; Pathological stage: {pathologic_stage}; T category: {pathologic_T}; N category: {pathologic_N}; M category: {pathologic_M}; Histological grade: {grade}.

Output: A single clinical narrative paragraph.

### Appendix 2 Representative clinical narratives and rule-based baseline

Example 1: complete structured clinical record.

Structured clinical input: Cancer type: HNSC; Age at diagnosis: 63; Sex: male; Pathological stage: Stage III; T category: T3; N category: N1; M category: M0; Histological grade: G2.

Generated clinical narrative: This is a 63-year-old male patient with head and neck squamous cell carcinoma. The tumor is classified as pathological Stage III, with T3 primary tumor extent, N1 nodal involvement, and no distant metastasis recorded as M0. The histological grade is G2.

Example 2: structured clinical record with missing variables.

Structured clinical input: Cancer type: BRCA; Age at diagnosis: 58; Sex: female; Pathological stage: Stage II; T category: T2; N category: unavailable; M category: M0; Histological grade: unavailable.

Generated clinical narrative: This is a 58-year-old female patient with breast invasive carcinoma. The tumor is classified as pathological Stage II, with T2 primary tumor extent. Nodal status is unavailable, and no distant metastasis is recorded as M0. The histological grade is unavailable.

For the rule-based CKP baseline in **Table 8**, the same structured variables were inserted into deterministic natural-language sentence templates without using an LLM. The template followed the same field order and missing-value rule as the LLM prompt, but used fixed sentence slots rather than model-generated wording. This baseline was used to evaluate whether the gain of CKP came from standardized clinical information alone or from the additional semantic normalization provided by LLM-generated narratives.
